# Effects of Aqueous Boundary Layers and Paracellular Transport on the Efflux Ratio as a Measure of Active Transport Across Cell Layers

**DOI:** 10.3390/pharmaceutics16010132

**Published:** 2024-01-19

**Authors:** Soné Kotze, Andrea Ebert, Kai-Uwe Goss

**Affiliations:** 1 Department of Analytical Environmental Chemistry, Helmholtz Centre for Environmental Research (UFZ), Permoserstraße 15, 04318 Leipzig, Germany; sone.kotze@ufz.de (S.K.); andrea.ebert@ufz.de (A.E.); 2 Institute of Chemistry, University of Halle-Wittenberg, Kurt-Mothes-Straße 2, 06120 Halle, Germany

**Keywords:** efflux transporters, MDCK, Caco-2, active transport, P-glycoprotein, permeability

## Abstract

The efflux ratio (ER), determined by Caco-2/MDCK assays, is the standard in vitro metric to establish qualitatively whether a compound is a substrate of an efflux transporter. However, others have also enabled the utilisation of this metric quantitatively by deriving a relationship that expresses the ER as a function of the intrinsic membrane permeability of the membrane ( $P_{0}$ ) as well as the permeability of carrier-mediated efflux ( $P_{pgp}$ ). As of yet, $P_{pgp}$ cannot be measured directly from transport experiments or otherwise, but the ER relationship provides easy access to this value if $P_{0}$ is known. However, previous derivations of this relationship failed to consider the influence of additional transport resistances such as the aqueous boundary layers (ABLs) and the filter on which the monolayer is grown. Since single fluxes in either direction can be heavily affected by these experimental artefacts, it is crucial to consider the potential impact on the ER. We present a model that includes these factors and show both mathematically and experimentally that this simple ER relationship also holds for the more realistic scenario that does not neglect the ABLs/filter. Furthermore, we also show mathematically how paracellular transport affects the ER, and we experimentally confirm that paracellular dominance reduces the ER to unity and can mask potential efflux.

## 1. Introduction

All transporter genes expressed in humans are divided into two major superfamilies known as the solute carrier (SLC) and ATP-Binding Cassette (ABC) transporters. As a general rule, the SLC family can be viewed as uptake transporters and the ABC family as efflux transporters [1]. In contrast to the SLC family, all of the transporters in the ABC family act as active transporters, and multidrug resistance (MDR) of cancerous tissue is largely attributed to the overexpression of this class of proteins due to their role in decreasing the intracellular concentration of cytotoxic compounds, including chemotherapeutic agents [2]. There are three major proteins associated with MDR that mediate efflux of drugs into the lumen and that exhibit broad substrate specificity: namely, P-glycoprotein (P-gp), Breast Cancer Resistance Protein (BCRP), and Multidrug Resistance-Associated Protein 2 (MRP2) [3]. Due to their central role in MDR and their potential to significantly impede the oral bioavailability of drugs, the study of efflux transporters remains a crucial point of interest in pharmaceutical research. However, despite the important role of these efflux transporters, “the science is not currently at a state where this can be routinely or reliably incorporated into physiologically based pharmacokinetic (PBPK) models” [4].

Within the ABC family, efflux facilitated by the P-gp transporter is the most widely observed and well-characterised [5]. Encoded for by the MDR1 (also known as ABCB1) gene, P-gp is embedded within the apical membrane of cells and, due to its remarkable promiscuity, is responsible for the active efflux of a wide range of structurally dissimilar drugs, many of which are clinically important [6]. As a result of its position and high expression in several pharmacological barriers such as the gastrointestinal tract (GIT) and blood–brain barrier (BBB), P-gp enhances the secretion of its substrates from these tissues, thereby diminishing its effective absorption and bio-availability [7,8].

Due to its pharmacokinetic importance, screening for transport via P-gp and other efflux transporters has become a crucial part of both the drug discovery and drug evaluation process [9]. In vitro cell systems have become essential for determining whether novel or existing drugs are substrates or inhibitors of important efflux transporters or whether there is significant risk of adverse drug–drug interactions [10]. Monolayer efflux studies that determine drug transport rates between an apical and basolateral compartment separated by a confluent cell monolayer grown on a permeable support are the most widely used and definitive of such systems. These bi-directional transport studies are particularly recommended for determining whether P-gp and/or BCRP interact with a drug of interest [11]. The most common cell types used in these assays are human colorectal adenocarcinoma (Caco-2) or Madin–Darby canine kidney (MDCK) cell lines, which are often modified with knock-out genes that inhibit the expression of other transporters or are transfected with genes that lead to the over-expression of the transporter of interest.

Transport studies performed with MDCK-MDR1 cells are generally used to evaluate P-gp activity for the drug under scrutiny, and this activity is evaluated by means of a metric called the efflux ratio (ER). Simply put, the ER is the ratio of secretory flux over absorptive flux, and, as we will show later, at its most basic level is essentially a measure of the relative contributions of active and passive transport to bi-directional flux across the monolayer. When it comes to the evaluation of drugs during development, guidance documents published by key regulatory agencies such as the Food and Drug Administration (FDA) and the European Medicines Agency (EMA) use the ER in a primarily qualitative manner. Often, establishing that the ER is greater than a threshold value of two is the sole requirement to achieve objectives such as: (i) ensuring the exclusion of drugs that are actively transported from biopharmaceutical classification systems (BCSs) for high-permeability drugs [12], (ii) evaluating whether further studies are recommended to determine which transporter(s) the drug is a substrate of [11], and (iii) investigating certain possible drug–drug interactions (DDI) that may alter absorption [13,14], in which case, further clinical studies are recommended. However, regulatory agencies also encourage the use of different threshold efflux ratios if it can be justified by prior experience or other reasoning.

Even though some of these guidelines also briefly advise on the prediction of potential DDI and absorption metrics with PBPK modelling, delving deeper into specific models reveals that the ER is often used as more than a guiding qualitative metric. For example, a study aiming to integrate in silico and in vitro tools uses the ER to optimise drugs against efflux facilitated by P-gp during drug development [15]. In many of these models, the ER is used as a significant input parameter [16]. Others aim to establish in vitro/in vivo correlations of the ER in order to promote the use of transport assays during drug discovery [17], and some even venture to use models to predict the ER from the molecular structures of the suspected substrates [18,19].

It is thus clear that the ER is seen as the predominant experimental measure for the occurrence of active transport. However, by its very nature, the ER cannot represent a so-called “intrinsic” value for active transport (as would be necessary for PBPK modelling) since this depends on the difference between active and passive transport. Indeed, Sugano et al. [20] derived the following expression for the ER:

(1) $$ER=\frac{P_{efflux}}{P_{PD}}+1,$$

where $P_{PD}$ is permeation via passive diffusion, and $P_{efflux}$ represents transport via carrier-mediated efflux. From this simple relationship, it is clear to see that the ER is a measure of the interplay between these two transport processes and not of active transport itself. As such, the aim of this work is to determine how an intrinsic value for the active transport of a substance via a transporter may be derived from permeability experiments. This intrinsic value for active transport would be analogous to the application of an intrinsic membrane permeability, $P_{0}$ ( $P_{PD}$ in Equation (1)), which is defined as a metric for the passive diffusion of the neutral species of a given compound through a membrane. The intrinsic nature of this value means that it would be independent of both pH and other transport processes, and since it is a measure of active transport through the membrane only, it naturally excludes artefacts such as any aqueous boundary layer (ABL) or filter.

However, Equation (1) was derived for a simple three-compartment model (apical compartment, cytosol, and basolateral compartment), and it is not entirely intuitive to assume that this simplistic relationship holds true for a model that represents the in vitro situation more closely by accounting for ABL and filter permeability as well as for paracellular transport. Without knowing whether the experimentally obtained ER truly represents only the two intrinsic values for passive and active transport, one cannot be sure the right values are extracted using this relationship.

This work thus deals with the quantitative effects of active transport and is conducted to probe the viability of an intrinsic value for carrier-mediated efflux and how our results can be generalised for all efflux transporters. Such a quantitative understanding is pivotal for optimising drug disposition and action. Furthermore, it is needed for in vitro–in vivo extrapolation (IVIVE) of transport parameters and for PBPK modelling, as the utilisation of such an intrinsic value would allow for the development of predictive methods that could simulate various transport scenarios for pharmaceutical compounds of interest.

The first step is to evaluate the integrity and robustness of ERs generated from monolayer assays, as any attempt to derive an intrinsic value from the ER could be confounded by ABL/filter limitation or paracellular transport. As such, our aims are (i) to determine what the possible effects are on the ER when the measured permeability of a compound is dominated by the ABL and/or the filter and (ii) to determine the influence of paracellular transport on the ER. In the theory section that follows, we will show how experimentally obtained permeability values from in vitro assays are a complex interplay of several individual transport resistances, and how the differences between the in vitro and in vivo scenarios necessitate the determination of intrinsic values.

## 2. Theory

### 2.1. In Vitro/In Vivo Extrapolation

There are various permeation barriers that a compound will encounter as it is passively and/or actively transported from the apical to basolateral compartment (A → B direction) and vice versa (B → A direction). The individual permeabilities of all permeation barriers contribute to the total apparent permeability ( $P_{app}$ ) that is measured in efflux assays. $P_{app}$ is measured separately in both the A→B and B→A directions. The relationship between flux and $P_{app}$ is as follows:

(2) $$P_{app}\;=\;\frac{C_{A,tx}-C_{A,tx-1}}{t_{x}-t_{x-1}}\;\times \;\frac{V_{A}}{A\;\times \;\Delta C},$$

where $\frac{C_{A,tx}-C_{A,tx-1}}{t_{x}-t_{x-1}}$ is the change in the cumulative concentration in the acceptor compartment per each elapsed time interval, $V_{A}$ (cm ${\text{​}}^{3}$ ) is the volume of the acceptor compartment, *A* (cm ${\text{​}}^{2}$ ) is the filter area, and ΔC ( μ g/mL) is the concentration difference between the donor and acceptor compartment calculated for each individual time step.

**Figure 1:**
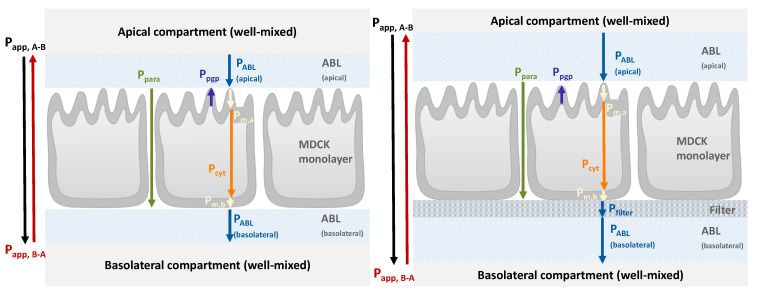
Permeation barriers and associated permeabilities in vivo and in vitro. In vivo, well-mixed donor and acceptor compartments are separated by an apical aqueous boundary layer (ABL), cell monolayer, and basolateral ABL. The in vitro system introduces a filter layer and a thicker ABL (not to scale). Adapted from Dahley et al. [23] to include active efflux facilitated by P-glycoprotein (P-gp). This is a simplified scheme that does not depict other processes that may occur in vivo, such as retention or metabolism.

To evaluate the potential of IVIVE, it is important to take note of and account for the differences between the in vitro and in vivo systems. Figure 1 shows that these two systems generally differ in two respects: namely, (i) the presence of the filter on which the cells are grown in in vitro assays and (ii) increased ABL thickness in in vitro systems [21].

For both the in vivo and in vitro scenarios, it is important to note that there are two parallel pathways that compounds can take in order to cross the monolayer [22]. Using the transcellular pathway (trans), chemicals pass through the membranes and cytosol of the cells. With the paracellular pathway (para), chemicals cross the monolayer through water-filled pores in the tight junctions between cells. Furthermore, Figure 1 also shows how experimentally derived $P_{app}$ measurements can be sub-divided to evaluate the relative influences of its constituent parts. In order to do this, each sub-process needs to be fully understood in order to be quantified. The relevant permeation barriers and their associated permeabilities that contribute to the measured $P_{app}$ will be discussed in detail in the following section, where we show how our model maps onto the in vitro scenario.

It is well-established that the hydrophobicity of a chemical affects its transport across cell monolayers. On a scale relative to pharmaceutical compounds, those with greater hydrophobicity (henceforth taken to mean compounds with a log hexadecane–water partition coefficient $logK_{hex/water}$ >−3 ; see Section 2.3 and Supplementary Materials) are quite membrane permeable and might have a $P_{m}$ value greater than the filter or ABL limit of the in vitro system, which means that the flux for such compounds will be limited by these layers. Moving in the opposite direction on this spectrum are those compounds that are more hydrophilic (henceforth taken to mean compounds with approximately $logK_{hex/water}<-6$ ; see Section 2.4 and Supplementary Materials) and are thus much less membrane permeable. These compounds may have a permeability even smaller than that of paracellular transport, which in turn also affects the measured flux. Because these two distinct effects tend to affect compounds far enough away from one another on the hydrophobicity spectrum, it is possible to split these two influences, enabling one to study them in isolation. For a good experimental system, both ABL/filter limitations and paracellular transport dominance are avoided, but in reality, the window of measurable $P_{app}$ is so small that it is often inevitable that either of these two factors affects flux in one or both directions. Because of this, it is crucial to study these influences, as it is only when all other sub-processes and their contributions are understood that we can hope to quantify $P_{pgp}$ .

### 2.2. Multi-Barrier Model

**Figure 2:**
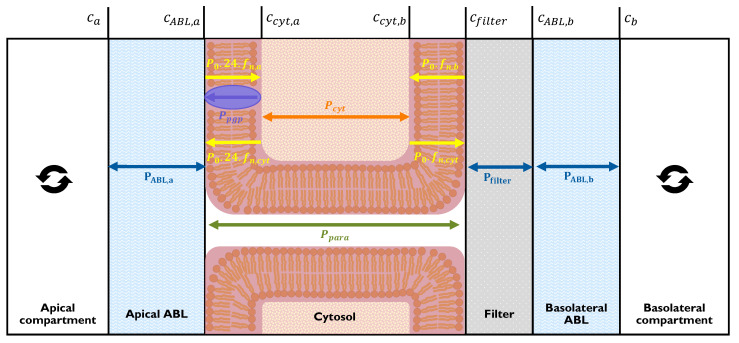
Permeation barriers, associated permeabilities, and concentrations in Caco-2 and MDCK transwell assays. Adapted from Dahley et al. [23] to include active efflux facilitated by P-gp. The well-mixed donor and acceptor compartments are separated by an apical ABL, cell monolayer, filter, and basolateral ABL. All concentrations shown are aqueous concentrations: $C_{a}$ is the bulk concentration on the apical side, $C_{ABL,a}$ is the ABL concentration on the apical side adjacent to the apical membrane, $C_{cyt,a}$ is the cytosolic concentration adjacent to the apical membrane, $C_{cyt,b}$ is the cytosolic concentration adjacent to the basolateral membrane, $C_{filter}$ is the filter concentration adjacent to the basolateral membrane, $C_{ABL,b}$ is the ABL concentration on the basolateral side adjacent to the filter, and $C_{b}$ is the bulk concentration on the basolateral side.

Figure 2 depicts the compartments and transport resistances that characterise our model approach and how our model maps onto the in vitro situation. $P_{x}$ represents the permeability of each respective barrier: namely, $P_{ABL,a}$ and $P_{ABL,b}$ are the permeabilities of the apical and basolateral ABLs, respectively; $P_{m,a}$ and $P_{m,b}$ are those of the apical and basolateral membranes, respectively; $P_{cyt}$ is that of the cytosol; and $P_{filter}$ is that of the filter. $P_{pgp}$ represents the permeability of P-gp mediated efflux from the cytosol to the apical compartment. It is important to note that transport by active efflux differs from passive transport not only by the unidirectional orientation but also by the fact that it is only driven by the substrate concentration at its binding site and not by a concentration gradient.

The total resistance (and thus permeability) of all the barriers depicted in Figure 2 is a function of each individual resistance found in series or parallel according to the natural laws of physics. In this case, there are two parallel resistances, which are the two different routes the chemical may use to cross the monolayer (para and trans). The ABLs and filter are serial resistances. Within the trans pathway where chemicals cross the cell, the P-gp resistance is in parallel with the resistance of the apical membrane, and the cytosol and basolateral membrane are once again found in series. These relationships between multiple serial and parallel resistances and how total flux is calculated from these principles can be found in Section 4.

$P_{m,a}$ , the permeability of the compound through the apical membrane, includes a factor of 24 to account for the microvilli, which increase the available surface area for permeation [24]. The aforementioned widely used metric for permeation via passive diffusion, known as intrinsic membrane permeability $P_{0}$ , is calculated from $P_{m,a}$ as shown in Equation (2) according to the pH–partition hypothesis, which postulates that only the neutral fraction ( $f_{n}$ ; see Supplementary Materials for the calculation) of a compound is able to pass through membranes.

(3) $$P_{m,a}=P_{0}\cdot f_{n}\cdot 24$$

There is no volume (and thus no storage capacity) assigned to the membranes, ABL, or cytosol, as these parts of the system are considered only as resistances. This is appropriate for experimental in vitro measurements, as the measurements are done under near steady-state conditions for which no change in storage occurs. Furthermore, any possible retention is accounted for by the use of a recovery-correction calculation described in Section 3.2.4. Furthermore, since P-gp is saturable, we assume concentrations low enough to ensure that the transporter is acting within its effective linear range.

Our measurements were done under ISO-pH conditions (i.e., the same pH in both the apical and basolateral compartments). Thus, we do not consider effects that may occur when a pH difference between the apical and basolateral compartments is applied, which will affect the transport of ionizable compounds. However, the full derivations in Supplementary Materials do include appropriate factors that are required to capture these so-called concentration-shift effects as described by Dahley et al. [23]. The effect of pH on the active efflux of the ionic or neutral species of an ionizing chemical will be the focus of an upcoming publication.

### 2.3. Special Considerations for the Evaluation of the ABL and Filter Effects

In addition to the general model assumptions outlined in Section 2.2, for the first aim of investigating the ABL and filter effects, there are other important considerations to take note of. Even though $P_{para}$ is included in the full derivations of the equations in Supplementary Materials, paracellular transport is ignored in this section, as the effect of this pathway on overall flux is negligibly small for neutral chemicals ( $logK_{hex/water}>-3$ ), which are expected to be affected by the ABL or filter limitation.

Aqueous permeation barriers (ABLs, cytosol, and water-filled pores of the filter) have been studied extensively and are well-understood [22,25]. $P_{ABL}$ is simply a function of the diffusion coefficient of the chemical in water and the thickness of the ABL. Likewise, $P_{cyt}$ can be calculated from the diffusion coefficient of the chemical in cytosol and the thickness of the cytosol. The calculation for $P_{filter}$ is similar to that of the ABL since it measures diffusion through water-filled pores; however, it is necessary to factor in the pore density and pore radius to calculate the surface area available for diffusion. Considering this, it is thus evident that $P_{ABL}$ can be decreased by increasing the thickness of the ABL, which can be achieved experimentally by not shaking the assay plates. Similarly, $P_{filter}$ can be decreased by using filters with a lower pore density. Using these principles as a starting point, for aim (i), we considered both the mathematical and experimental implications of ABL and/or filter dominance on the ER. Thus, MDCK monolayer efflux studies were performed using compounds for which such experimental ABL and/or filter variations are expected to affect the measured flux in one or both directions in order to evaluate the impact this has on the ER.

### 2.4. Special Considerations for the Evaluation of Paracellular Transport

For the second aim of investigating the effect of paracellular transport, all general model assumptions outlined in Section 2.2 hold true; however, in contrast to Section 2.3, $P_{para}$ is explicitly included in the model in order to quantify the effect of this pathway on overall flux for chemicals with a $logK_{hex/water}$ less than roughly −6 , which are expected to preferentially take the para route. This threshold was also determined by looking at the existing literature [23,26]. However, it is by no means a strict threshold, as there are some uncertainties. $P_{para}$ can vary depending on the compound, and experimental and modelling results place the $logK_{hex/water}$ of affected compounds anywhere from <−5 and lower.

For aim (ii) we considered the mathematical implications of paracellular dominance on the ER. To validate these mathematical conclusions, we present a small subset of data from MDCK monolayer efflux studies performed for an upcoming publication. These sample data represent assays performed for compounds at external pH values for which paracellular transport is expected to dominate the measured flux in both directions in order to demonstrate the impact this has on the ER.

## 3. Materials and Methods

### 3.1. Cells

#### 3.1.1. Cell Selection

The MDCK-MDR1 cell line was generated by transfecting MDCK cells with the human mdr1 gene [27], which leads to over-expression of human P-gp on the apical side of the polarised cell monolayer, making this the preferred in vitro model for P-gp in the human intestinal mucosa. As such, for this study, MDCK-II-MDR1 cells transfected with human MDR1 for P-gp expression were obtained from The Netherlands Cancer Institute (Amsterdam, The Netherlands).

#### 3.1.2. Cell Culture

The cell medium was Dulbecco’s modified Eagle medium (DMEM) (1X) + GlutaMAX™-I supplemented with 10% FBS and 100 U/mL penicillin and 100 µg/mL streptomycin. Cells were maintained at 37 °C in an atmosphere of 5% CO ${\text{​}}_{2}$ and passaged twice a week. All chemicals and suppliers can be found in Supplementary Materials.

### 3.2. Monolayer Efflux Studies

#### 3.2.1. Selection and Preparation of Test Compounds

Candidate compounds for bidirectional transport studies were selected based on collections of identified P-gp substrates with efflux ratios > 2 as determined by MDCK-MDR1 transport experiments and according to their $logK_{hex/water}$ as predicted by the UFZ LSER database [28]. Zwitterions were excluded. For aim (i), ABL-limited compounds for which paracellular transport may affect $P_{app}$ values were also excluded, and based on these criteria as well as the solubility and detectability by LC/MS, the compounds dipyridamole ( $logK_{hex/water}$ :−4.89), quinidine ( $logK_{hex/water}$ :0.14), and loperamide ( $logK_{hex/water}$ :−0.45) were selected. For aim (ii), the compound doxorubicin ( $logK_{hex/water}$ :−8.86) was used, as the para route was calculated to be dominant in both directions at a determined external pH value.

#### 3.2.2. Bi-Directional Transport Experiments

For transport experiments, MDCK-MDR1 cells were used between passages 20 and 40. Cells were seeded onto 12-well PET inserts (CellQART, Northeim, Germany; pore size: 0.4 µm; filter thickness: 11.5 µm) at a density of 1.5 × $10^{5}$ cells/insert. Inserts with a high porosity of 100 × $10^{6}$ pores/cm ${\text{​}}^{2}$ or low porosity of 2 × 10 ${\text{​}}^{6}$ pores/cm ${\text{​}}^{2}$ were used. After seeding, cells were maintained as described in Section 3.1.2 for 4 days to ensure confluent cell monolayer formation. The cell medium was exchanged one day before the transport experiments were performed. Prior to the transport experiments, the inserts were washed with Hank’s balanced salt solution (HBSS) to remove residual DMEM. Where inserts were used for the determination of $P_{0}$ , they were pre-incubated for 30 min in HBSS pH 7.4 with 2 µM elacridar (a known P-gp inhibitor) to prevent active transport. For all other inserts, no inhibitor was used.

The apical-to-basolateral ( A→B direction) as well as the basolateral-to-apical ( B→A direction) transport rates of the test compounds were determined in duplicate with varying stirring and filter conditions. The three conditions tested were as follows: high-porosity filters, stirred (HPS); high-porosity filters, unstirred (HPU); and low-porosity filters, stirred (LPS). The transport buffer was HBSS with 25 mM HEPES at pH 7.4. Stock solutions were prepared in the transport buffer, and all solutions were pre-warmed to 37 °C. Additional stock solutions with 2 µM elacridar were prepared at different pH values to measure $P_{0}$ values in tandem with the standard experiments. Experiments for each compound also included a reference compound to ensure and confirm consistent P-gp expression and/or activity. Details of these additional experiments are listed in Supplementary Materials. The pHs of the buffer and stock solutions prior to the experiment as well as the pHs of all samples after experiment completion were controlled with a rapid pH automated pH meter (Hudson Robotics, Inc., Springfield, NJ, USA). The pHs remained within a range of ±0.15.

Inserts were used in 12-well plates (TPP Techno Plastic Products AG, Trasadingen, Switzerland), the basolateral compartment volume was 1.6 mL, and the apical compartment volume was 0.5 mL. The transport experiments were initiated by replacing the HBSS with transport buffer and the test compound solution.

For measurement of transport in the A→B direction: Inserts were placed in 12-well plates containing transport buffer in the basolateral compartment, after which, the test compound solution was added to the apical compartment. At every sampling step, each insert was placed into a new well containing fresh, warmed assay buffer, and the old buffer was sampled. Sampling occurred at four consistent time intervals.

For measurement of transport in the B→A direction: Inserts were placed in 12-well plates containing the test compound solution in the basolateral compartment (donor), after which, transport buffer was added to the apical compartment. At each sampling time step, 300 μ L was sampled from the apical compartment (the maximum amount that could be removed without disturbing the cell layer) and replaced with an equal volume of warmed, fresh transport buffer. Sampling occurred at four consistent time intervals. Time intervals for sampling were determined uniquely for each compound and direction measured to ensure that sink conditions would be maintained throughout the experiment. After the initiation of the experiments and between sampling steps, the plates were placed in an orbital shaking incubator at 450 rpm and 37 °C (Titramax and Inkubator 1000, Heidolph Instruments GmbH & Co. KG, Schwabach, Germany) with the exception of the HPU experiment plates, which were only incubated at 37 °C (Heraeus HERAcell 150 CO ${\text{​}}_{2}$ Incubator, Thermo Electron LED GmbH, Langenselbold, Germany) without any shaking. For both directions, the donor compartment was also sampled (i.e., the apical compartment for A→B and the basolateral compartment for B→A ) at the final timestep in order to determine the recovery.

#### 3.2.3. Monolayer Integrity Assessment

The uniformity of cell monolayer growth between inserts was assessed to ensure comparability of the results. Immediately prior to and after the completion of each transport experiment, the TEER across the MDCK-MDR1 monolayer was measured for each insert at 37 °C at three positions using an EVOM epithelial tissue volt/ohmmeter (World Precision Instruments Inc., Sarasota, FL, USA). The average TEER of the HP inserts was 136 ± 7 Ω cm ${\text{​}}^{2}$ before and 129 ± 5 Ω cm ${\text{​}}^{2}$ after the transport experiments, and the average TEER of the LP inserts was 160 ± 8 Ω cm ${\text{​}}^{2}$ before and 150 ± 6 Ω cm ${\text{​}}^{2}$ after the transport experiments, thereby confirming the integrity of the cell monolayers throughout the experiment.

In addition to the TEER measurement, Lucifer yellow (LY) was used as a marker in the transport assays, which were performed to confirm the integrity of the cell monolayer grown on each insert. After the final sampling step of the transport experiment, each insert was placed in well plates with fresh transport buffer in the basolateral compartment. LY stock solution (100 μ g/mL, diluted in transport buffer) was added to the apical compartment after discarding the remaining volume. The plates were incubated for 60 min in the orbital shaker (450 rpm, 37 °C), after which, the fluorescence intensity (Ex: 485, Em: 538) of samples from the basolateral compartment was measured in a 96-well microplate using a SpectraMAX Gemini EM spectrophotometer (Molecular Devices LLC., San Jose, CA, USA). If the LY permeability of any insert was found to be above the pre-defined threshold of 1.5 × $10^{-6}$ cm/s, the result was to be excluded [29]. However, only for 3 out of 36 inserts did the $P_{app}$ of LY slightly exceed the threshold, but the derived $P_{app}$ results for the test compounds of those inserts were qualitatively similar to those determined with the replicate inserts within the LY threshold. Based upon responsible scientific judgment, the cell monolayer was considered acceptable, and the results from these inserts were included in the final results.

#### 3.2.4. Sample Analysis and Calculations

Samples were analysed with an Infinity II 1260 LC system coupled with a 6420 triple quadrupole 145 with ESI source (Agilent Technologies Inc., Santa Clara, CA, USA). A Kinetex^®^ C18 (2.6 µm; 100 Å; 50 ∗ 3.0 mm) LC column was used (Phenomenex Inc., Torrance, CA, USA). Gradient elution was performed with double-distilled water (1% MeOH and 0.1% HCOOH; pH 2.7) as well as MeOH (0.1% HCOOH), which were used as the aqueous and organic eluents, respectively.

For both directions, $P_{app}$ was calculated from the acceptor compartment concentrations $C_{A}$ measured for at least three consecutive timepoints according to Equation (2). $P_{app}$ values determined at each timestep were corrected with the calculated recovery for that monolayer as done by Neuhoff [30]. Data are presented as the mean of the recovery-corrected $P_{app}\;\pm$ standard deviation of at least three timestep samples of both replicates. The first timestep in the A→B direction was excluded in order to account for lag time [31]. The ER was calculated as the ratio of these mean $P_{app}$ values in the B→A direction and A→B direction as in Equation (4) for each experimental condition (HPS, HPU, and LPS).

(4) $$ER=\frac{P_{app,B\to A}}{P_{app,A\to B}}$$

#### 3.2.5. Paracellular Transport Measurement

Bi-directional transport experiments were performed as described in Section 3.2, however, only for standard (i.e., HPS) conditions and using an external buffer pH (apical and basolateral) of 6 and 7.

## 4. Results and Discussion

### 4.1. Mathematical Implications of the ABL for Initial Absorptive and Secretory Flux Rates

As previously described, ignoring paracellular transport and assuming an ISO-pH method, the mathematical treatment of the scenario for which the ABL is included (see Figure 2) results in the following expression for the steady-state flux in the A→B direction (absorptive) under constant donor concentrations and infinite sink conditions ( $C_{b}=0$ ):

(5) $$J_{A\to B}=\frac{1}{\frac{1}{P_{ABL,a}}+\frac{1}{P_{trans,A\to B}}+\left(1+\frac{P_{pgp}}{P_{0}\cdot 24\cdot f_{n,cyt}}\right)\cdot \left(\frac{1}{P_{filter}}+\frac{1}{P_{ABL,b}}\right)}\cdot C_{a},$$

where:

(6) $$P_{trans,A\to B}=\frac{1}{\frac{1}{P_{0}\cdot 24\cdot f_{n,a}}+\left(1+\frac{P_{pgp}}{P_{0}\cdot 24\cdot f_{n,cyt}}\right)\cdot \left(\frac{1}{P_{cyt}\cdot \frac{f_{n,a}}{f_{n,cyt}}}+\frac{1}{P_{0}\cdot f_{n,a}}\right)}$$

Here, $C_{b}$ refers to the concentration in the acceptor (i.e., basolateral) compartment and $C_{a}$ to the concentration in the donor (i.e., apical) compartment. From the above expressions and the physical principles of serial and parallel resistances described in Section 2.2, it is clear that all passive permeabilities are symmetric and influence the flux as serial resistances. In addition, from Equations (5) and (6), one can see that the apical membrane and ABL are unaffected by $P_{pgp}$ , but that $P_{pgp}$ can influence all resistances found downstream from the P-gp transporter. It can also be seen that each single resistance, including $P_{pgp}$ , can completely dominate the absorptive flux if it is large enough. This means that $P_{pgp}$ can wholly minimise flux in the A→B direction (once again, under the assumption of negligible paracellular transport).

For the steady-state flux in the B→A direction (secretory) under constant donor concentrations and infinite sink conditions ( $C_{a}=0$ ), the following expression is obtained:

(7) $$J_{B\to A}=\frac{1}{\frac{1}{P_{ABL,b}}+\frac{1}{P_{filter}}+\frac{1}{P_{trans,B\to A}}+\frac{1}{P_{ABL,a}}/\left(1+\frac{P_{Pgp}}{P_{0}\cdot 24\cdot f_{n,cyt}}\right)}\cdot C_{b},$$

where:

(8) $$P_{trans,B\to A}=\frac{1}{\frac{1}{P_{0}\cdot 24\cdot f_{n,a}}+\left(1+\frac{P_{pgp}}{P_{0}\cdot 24\cdot f_{n,cyt}}\right)\cdot \left(\frac{1}{P_{cyt}\cdot \frac{f_{n,a}}{f_{n,cyt}}}+\frac{1}{P_{0}\cdot f_{n,a}}\right)}\cdot \left(1+\frac{P_{pgp}}{P_{0}\cdot 24\cdot f_{n,cyt}}\right)$$

Here, $C_{a}$ refers to the concentration in the acceptor (i.e., apical) compartment and $C_{b}$ to the concentration in the donor (i.e., basolateral) compartment. In contrast to the absorptive flux, the influence of active efflux by P-gp can never dominate the secretory flux as a whole but can at best level out the secretory flux resistance in the apical membrane and ABL so that the total secretory flux is then governed by the basolateral resistance. Note that Equations (5) and (7) reduce to the flux through the serial resistances of all permeation barriers when $P_{pgp}$ is zero, as one should expect when a chemical permeates via passive diffusion alone. Full derivations of the above expressions for absorptive and secretive flux can be found in Supplementary Materials.

### 4.2. Mathematical Implications of the ABL for the ER

As described earlier, the ER is generally defined as the quotient of the steady-state secretory and absorptive flux rates under sink conditions and with constant donor concentrations. That is:

(9) $$ER\equiv \frac{J_{B\to A}}{J_{A\to B}}$$

For the system depicted in Figure 2 and with the assumption of no pH gradient, if the full flux Equations (5) and (7) are substituted into Equation (9), the following expression is derived for the ER as a function of the permeability of the apical membrane $\left(P_{m,a}=P_{0}\cdot 24\cdot f_{n,cyt}\right)$ and the so-called intrinsic permeability of the P-gp transporter $\left(P_{pgp}\right)$ :

(10) $$ER=\frac{P_{pgp}}{P_{m,a}}+1$$

This surprisingly simple expression for the ER indicates that the resistances in the cytosol, the basolateral membrane, the filter, and both ABLs cancel out in their effects on the ER. This shows that even for our more explicit model, the relationship (Equation (1)) that Sugano et al. obtained [20] for a simplistic, less realistic three-compartment model holds true. The fact that this simple equation is also valid for the more representative model approach depicted in Figure 2 is by no means intuitive. At first glance, it may seem that adding the ABL to the model is just the straightforward addition of another passive resistance that could even be lumped together with membrane resistance. However, it is imperative to consider the ABL as a resistance separate from the membrane because of two very consequential reasons. Firstly, there are two ABLs that need to be considered. While the basolateral ABL resistance can, in theory, be lumped together with that of the basolateral membrane (as long as fractionation factors are accounted for), the same cannot be done with the apical ABL due to the presence of the P-gp transporter in the apical membrane. Since P-gp only spans the membrane, its effect does not extend to the apical ABL. As such, the apical membrane is not simply a passive resistance layer to which other passive resistances can be added. However, despite these considerations, which result in a considerably more complex mathematical scenario, the ER equation reduces to the same simple relationship for the ISO-pH method. In this case, it is evident that the ER solely depends on the relative influence of active transport and the passive resistance in the apical membrane (or, more precisely, the passive resistance that runs parallel to the active transporter). Secondly, passive permeability through the ABL differs from that of the membrane since the total concentration of the compound passes through the ABL, not just the neutral fraction. This has direct consequences for the ER of ionizable compounds if the gradient method is used (see Supplementary Materials, Equation (S19)). Therefore, affirming this relationship renders the ER a very meaningful parameter for the quantification of intrinsic information of active efflux provided that $P_{para}$ is negligible and there is no pH gradient.

From Equation (2), for $P_{0}$ , it is clear that when the $pK_{a}$ of a compound and the external pH are known, then the intrinsic value for membrane permeation via passive diffusion can be easily determined by in vitro transport experiments, provided that the membrane is the dominating resistance and an inhibitor is used to rule out active transport [23,25]. Thus, crucially, experimental ER values provide easy access to the proposed intrinsic values of $P_{pgp}$ if $P_{m,a}$ is known. In comparison, if $P_{pgp}$ had to be determined from either the absorptive or the secretory flux, it would be significantly more error-prone, as uncertainties in the permeabilities of both ABLs and the filter (instead of cancelling out) would also come into play.

Furthermore, Equation (10) reveals that an ER value not significantly larger than one (measured with the ISO-pH method) is an unambiguous indication that no carrier-mediated transport occurs, provided that paracellular transport can be ruled out and provided that the drug concentrations are low enough to exclude any saturation effect on the transporter. ER measurements by these transport assays can thus be seen as a reliably definitive way to detect carrier-mediated drug efflux.

### 4.3. Experimental Implications for the ER When the ABL or Filter Dominates Flux

In order to corroborate the conclusions drawn from the mathematical treatment described in Section 4.1 and Section 4.2, we aimed to investigate experimentally whether the ER is independent of both ABL and filter resistances. To do this, we performed experiments for which the relative influences of these resistances were varied. Experiments were performed under three conditions wherein either a high- or low-porosity filter was used to vary $P_{filter}$ and wherein assay plates were shaken or not in order to vary ABL thickness. The three different experimental conditions are labelled as follows: HPS—high-porosity filter, stirred; LPS—low-porosity filter, stirred; HPU—high-porosity filter, unstirred. The $P_{app}$ values measured in these assays for each respective condition in both directions as well as the resultant ER values are depicted in Table 1.

Table 1 shows that $P_{app}$ values (i.e., individual fluxes in both directions) determined under these varying experimental conditions differ substantially. For relatively hydrophobic chemicals such as these, this is to be expected, as the flux in one or both directions is dominated by the ABL or filter resistance under one or more of the three experimental conditions (HPS, HPU, and LPS), and thus, the $P_{app}$ determined in either direction is a measure of $P_{ABL}$ or $P_{filter}$ as it is varied. This corroborates our findings in Section 4.1 that aqueous permeation barriers do indeed affect absorptive and secretory flux, as it is evident that $P_{app}$ values differ by up to a magnitude of 10 for the selected compounds when these barriers are experimentally manipulated. Crucially, however, even with these large differences in $P_{app}$ between measured directions and experimental conditions, the ER does not fluctuate as considerably, and it, in fact, stays relatively stable. The mean ERs obtained for quinidine and loperamide were greater than two but were still relatively low. Thus, even though this still qualifies them as P-gp efflux substrates and the results corroborate our findings, data from these low-ER compounds are not considered as demonstrative as those of dipyridamole.

The results depicted in Table 1 validate our mathematical findings in Section 4.2 that the effects of the ABL and filter are cancelled out and have no bearing on the ER. In other words, the ER is independent of filter and ABL resistance. This finding is very consequential for ER assays, as it indicates that when hydrophobic chemicals are under investigation, one does not need to be concerned with measures (whether stirring, pH manipulation, etc.) to ensure that compounds are measured above the ABL or filter limitation, as is crucial when one attempts to determine $P_{m}$ .

### 4.4. Mathematical Implications of Paracellular Transport on the ER

In the above sections, we investigated the effects of the ABL and/or filter, which affect measured flux for relatively hydrophobic chemicals. For these chemicals, paracellular transport is insignificant enough to be ignored in the calculations. However, when performing efflux assays with more hydrophilic chemicals, paracellular transport may become significant and thus needs to be explicitly considered in the absorptive and secretory flux equations and, consequently, in the ER equation. Full derivations for $J_{A\to B}$ and $J_{B\to A}$ and the ER including $P_{para}$ can be found in Supplementary Materials.

Of particular note is the special case that occurs when $P_{para}$ dominates in both transport directions, and it is found that ER = 1. In contrast to our findings above, this result is quite intuitive. Since paracellular transport is symmetric, it can be deduced that the ER would approach unity if paracellular transport dominates flux in both directions. However, this means that when the compound is primarily transported via the para route, as is the case with more hydrophilic compounds, then carrier-mediated transport may be masked. As a result, when these assays are performed with chemicals for which $P_{para}$ dominates, it may be incorrectly assumed that no efflux occurs because an ER of one is obtained, but in reality, transport may be occurring that simply cannot be quantified as a consequence of this dominance and its effect on the ER.

Evidently, in contrast to ABL limitation, paracellular transport does have an effect on the ER, and depending on the compound, the effect can be significant enough to reduce the ER to unity, indicating the absence of efflux. This, of course, makes any meaningful determination of intrinsic values impossible when paracellular transport is dominant. As a result, it appears that while it is not necessary to take measures to avoid ABL or filter limitations, it is indeed necessary to avoid complete paracellular transport dominance or to account for the paracellular contribution when calculating the ER using the relevant equations. Just exactly how much paracellular transport affects the ER when it is not dominant yet contributes to flux through the monolayer needs to be calculated individually for each chemical. However, these calculations are, of course, heavily dependent on some estimated value of $P_{para}$ . This is important because, in the past, it has been assumed that $P_{para}$ is negligible [20].

### 4.5. Experimental Implications of Paracellular Transport on the ER

Table 2 shows a sample of data of monolayer efflux assays performed for an upcoming publication. Assays for doxorubicin ( $pK_{a}$ = 9.56) were performed at various external pH values (ISO-pH method), and Table 2 represents the data from assays performed at pH 6 and pH 7. At pH 6, paracellular transport of doxorubicin is expected to dominate the measured flux in both directions, and from the $P_{app}$ values for pH 6, one can see that both A→B and B→A flux are close to the assumed values for paracellular transport, and the resultant ER is close to one, just as the mathematical findings in Section 4.4 predict. In contrast, at pH 7 when the neutral fraction is increased, it seems that flux in the B→A is no longer dominated by paracellular transport, and the $P_{app}$ increases. As a result, the ER rises above the >2 threshold, signalling significant P-gp efflux.

These experimental results corroborate the conclusions drawn above from the mathematical treatment of a scenario for which paracellular transport dominates the flux in both directions and once again highlights the importance of considering paracellular transport when performing bidirectional transport studies. It is clear that complete paracellular dominance in an in vitro system can mask any efflux that may be occurring, and the resultant ER of one can lead to the false classification of compounds as non-substrates.

## 5. Conclusions

Equation (10) has been derived by others in an attempt to utilise the ER in a quantitative manner, and it provides easy access to $P_{pgp}$ as a more suitable measure of active transport. Thus, this simple relationship paves the way for deriving intrinsic permeability values for active efflux, which are essential for reliable PBPK modelling from experimentally obtained ERs and $P_{0}$ values. However, until now, this relationship had only been proven for a simpler system that neglects the transport resistances of the ABL and filter as well as paracellular transport. It is known that individual fluxes in either direction are heavily affected by ABL limitation, so it is crucial to investigate any possible effects on the ER. We present a model that includes the apical and basolateral ABLs as well as the filter; our mathematical findings and experimental data both show that the ER is independent of ABL or filter limitations, and we prove that Equation (10) remains valid for a more realistic scenario that considers these in vitro aqueous barriers. As a consequence, bidirectional transport assays performed to determine the ER of a substrate need not be concerned with the often painstaking and time-consuming measures taken to avoid ABL/filter limitations as with assays performed to determine $P_{0}$ , nor do existing ER data have to be re-evaluated due to ABL interference. In contrast, our mathematical and experimental results show that paracellular transport can have a significant effect on the ER. Indeed, paracellular transport can affect the ER even when it is not the dominant transport route, and thus, its role must always be considered when interpreting experimental results. Furthermore, when paracellular transport dominates in both measured directions, then the ER reduces to unity. Determination of the ER when paracellular transport dominates can incorrectly classify a compound as a non-substrate. Yet efflux from the cytosol may be occurring. Though we have described the model for P-gp in this study, the model is generalisable to any efflux transporter in the apical membrane. Furthermore, it is important to note that the model and ER relationship presented in this study are derived for the ISO-pH method. Deviations from the model will occur if there are other significant processes that are not accounted for, such as the presence of additional transporters in the basolateral membrane. However, the model can be extended to include such scenarios. In an upcoming publication, we will investigate the model and ER relationship for charged species at various pH values and will evaluate and validate the model by fitting it to further experimental data.

## Figures and Tables

**Table 1:** $P_{app}$ values in the A→B and B→A directions ± standard deviation. $P_{app}$ represents the mean of at least three timepoints and two replicates (n = 2). ER represents the quotient of $P_{app,B\to A}$ over $P_{app,A\to B}$ . HPS: High-porosity filter, stirred. LPS: Low-porosity filter, stirred. HPU: High-porosity filter, unstirred.

		$P_{\mathrm{app},A\to B}$ (cm/s) $\times \;10^{-6}$	$P_{\mathrm{app},B\to A}$ (cm/s) $\times \;10^{-6}$	ER	Recovery (%)
Dipyridamole (12 μ M)	HPS	4.03 ± 0.28	97.2 ± 13.9	24.2 ± 4.1	91.0–108
	LPS	0.38 ± 0.06	7.20 ± 1.30	19.8 ± 5.7	90.3–114
	HPU	2.71 ± 0.42	52.5 ± 8.77	19.3 ± 1.5	78.9–90.9
Quinidine (12 μ M)	HPS	65.2 ± 6.32	145 ± 8.26	2.2 ± 0.13	73.7–98.5
	LPS	12.4 ± 0.07	24.9 ± 0.87	2.0 ± 0.11	78.9–100
	HPU	27.0 ± 5.65	52.3 ± 9.75	2.0 ± 0.26	76.8–121
Loperamide (10 μ M)	HPS	46.7 ± 5.03	126 ± 27.8	2.7 ± 0.73	75.0–88.1
	LPS	5.65 ± 0.42	15.9 ± 4.24	3.2 ± 0.91	69.2–86.1
	HPU	9.80 ± 2.84	29.2 ± 3.31	2.8 ± 0.87	62.3–81.3

**Table 2:** Recovery-corrected $P_{app}$ values in the A→B and B→A directions ± standard deviation for doxorubicin at pH 6 and pH 7. $P_{app}$ represents the mean of 3 timepoints and 3 replicates (n = 3). ER represents the quotient of $P_{app,B\to A}$ over $P_{app,B\to A}$ .

	${\mathrm{pH}}_{\mathrm{ext}}$	$P_{\mathrm{app},A\to B}$ (cm/s) $\times \;10^{-6}$	${\mathrm{logP}}_{\mathrm{app},A\to B}$	$P_{\mathrm{app},B\to A}$ (cm/s) $\times \;10^{-6}$	${\mathrm{logP}}_{\mathrm{app},B\to A}$	ER	Recovery (%)
Doxorubicin	6	0.14 ± 0.04	−6.85	0.23 ± 0.08	−6.93	1.6	89.3–95.3
	7	0.12 ± 0.03	−6.64	0.78 ± 0.36	−6.11	6.5	87.8–93.1

## Data Availability

The data presented in this study are available upon request from the corresponding author.

## Supplementary Materials

The following supporting information can be downloaded at: www.mdpi.com/xxx/s1, Table S1: List of mathematical abbreviations; Table S2: List of chemicals and suppliers; Table S3: $P_{app}$ and ER values for the reference compound; Table S4: Experimental details, $P_{app}$ and calculated ABL thickness; Table S5: $P_{app}$ with inhibitor, ER and calculated $logP_{0}$ for measured compounds. References [32,33,34,35,36,37] are cited in the Supplementary Material.
