# Supplementary material for: Effects of Aqueous Boundary Layers and Paracellular Transport on the Efflux Ratio as a Measure of Active Transport Across Cell Layers
Source: Pharmaceutics. 2024 Jan 19;16(1):0. doi: 10.3390/pharmaceutics16010132 (PMC11154460; doi:10.3390/pharmaceutics16010132)
Supplement: Supplementary file 1 [file pharmaceutics-16-00132-s001.zip › pharmaceutics-2770991-supplementary.pdf]

Supplementary Material

Effects of aqueous boundary layers and paracellular transport on the efflux ratio as a measure for active transport across cell layers

Soné Kotze, Andrea Ebert, and Kai-Uwe Goss

I Mathematical derivations of Equations 5–10

Table S1: List of mathematical abbreviations

Abbreviation	Description	Reference
C_a	Bulk concentration on the apical side	
$C_{ABL,a}$	ABL concentration on the apical side adjacent to the apical membrane	
$C_{ABL,b}$	ABL concentration on the basolateral side adjacent to the filter	
C_b	Bulk concentration on the basolateral side	
$C_{cyt,a}$	Cytosolic concentration adjacent to the apical membrane	
$C_{cyt,b}$	Cytosolic concentration adjacent to the basolateral membrane	
C_{filter}	Filter concentration adjacent to the basolateral membrane	
x	Thickness of the resistance layer	
D_{cyt}	Diffusion coefficient through cytosol: $D_w \cdot 0.05$	Calculation according to Verkman et al. [32]
D_w	Diffusion coefficient through water: $D_W = 1.348 \cdot 10^{(-4.13-0.453 \cdot \log MW)}$	Calculation according to Avdeef et al. [26]

ER	Efflux ratio: $P_{app,B\rightarrow A}/P_{app,A\rightarrow B}$	
$f_{n,cyt}$	Fraction of neutral species in the cytosol	Calculation according to Dahley et al. [23]
$f_{n,a}$	Fraction of neutral species on the apical side	Calculated according to [33–35]
$f_{n,b}$	Fraction of neutral species on the basolateral side	
$P_{app,A\rightarrow B}$	Experimentally obtained apparent permeability in the apical to basolateral direction	
$P_{app,B\rightarrow A}$	Experimentally obtained apparent permeability in the basolateral to apical direction	
$P_{ABL,a}$	Permeability of both species through the apical ABL: $P_{ABL,a} = D_w/x_{ABL,a}$	
$P_{ABL,b}$	Permeability of both species through the basolateral ABL: $P_{ABL,b} = D_w/d_{ABL,b}$	
P_{cyt}	Permeability of both species through the cytosol: $P_{cyt}=D_{cyt}/x_{cyt}$	[22]
P_{filter}	Permeability of both species through the filter	Porosity calculated according to [36]
$P_{m,a}$	Permeability of the neutral species through the apical membrane: $PS_{m,a}= P_0*24*f_n$	Factor 24 to account for microvilli [24]
$P_{m,b}$	Permeability of the neutral species through the basolateral membrane: $PS_{m,b}= P_0*f_n$	
P_0	Intrinsic membrane permeability of the neutral species	
P_{para}	Paracellular transport, assumed equal in both directions	
P_{pgp}	Intrinsic permeability of carrier-mediated efflux	

$P_{trans,A\rightarrow B}$	Apparent transcellular permeability, including active transport, diffusion across membranes, and diffusion across the cytosol, in A→B direction	
$P_{trans,B\rightarrow A}$	Apparent transcellular permeability, including active transport, diffusion across membranes, and diffusion across the cytosol, in B→A direction	
$S_{ABL,a}^{A\rightarrow B}$	Concentration shift effect in apical ABL in the basolateral to apical direction	[23]
$S_{ABL,b}^{B\rightarrow A}$	Concentration shift effect in basolateral ABL and Filter in the apical to basolateral direction	[23]
$S_{cyt}^{A\rightarrow B}$	Concentration shift factor in the cytosol in the apical to basolateral direction	[23]
J	Steady-state flux	

For the following derivations, it is assumed that the passive permeation of the ionic species through the biological membranes is negligible. Permeation through aqueous films is assumed to be the same for all species, regardless of the ionisation state of the compound. The fluxes J (unit: mass/area/time) in the individual compartments of the system described in Figure 2 are described as follows:

$$J_{ABL,a} = P_{ABL,a}(C_a - C_{ABL,a}) \quad (S1)$$

$$J_{cell} = J_{trans} + J_{para} = P_{trans,A\rightarrow B} * C_{ABL,a} - P_{trans,B\rightarrow A} * C_{filter} + P_{para} * (C_{ABL,a} - C_{filter}) \quad (S2)$$

$$J_{filter} = P_{filter}(C_{filter} - C_{ABL,b}) \quad (S3)$$

$$J_{ABL,b} = P_{ABL,b}(C_{ABL,b} - C_b) \quad (S4)$$

At steady state, the flux is the same through each compartment:

$$J_{A\rightarrow B} = J_{ABL,a} = J_{cell} = J_{filter} = J_{ABL,b} \quad (S5)$$

The individual fluxes in the compartments comprising the cellular layer are described as follows:

$$J_{m,a} = C_{ABL,a} * f_{n,a} * P_0 * 24 - C_{cyt,a} * \left(f_{n,cyt} * P_0 * 24 + P_{pgp}\right) \quad (S6)$$

$$J_{cyt} = P_{cyt} * \left(C_{cyt,a} - C_{cyt,b}\right) \quad (S7)$$

$$J_{m,b} = P_0\left(f_{n,cyt} * C_{cyt,b} - f_{n,b} * C_{filter}\right) \quad (S8)$$

At steady state, the flux is the same through each cellular compartment:

$$J_{trans} = J_{m,a} = J_{cyt} = J_{m,b} \quad (S9)$$

As a consequence of the above flux equations, the permeability through the cell layer in the A ⟶ B direction can be described as follows (a more detailed derivation for P_{trans} from S6–S9 can be found in Section VI):

$$P_{trans,A\rightarrow B} = \frac{1}{\left(1 + \frac{P_{pgp}}{P_0 * 24 * f_{n,cyt}}\right) * \left(\frac{1}{f_{n,a} * P_0} + \frac{1}{\frac{f_{n,a}}{f_{n,cyt}} * P_{cyt}}\right) + \frac{1}{P_0 * 24 * f_{n,a}}}$$

$$= \frac{1}{\left(1 + \frac{P_{pgp}}{P_0 * 24 * f_{n,cyt}}\right) * \left(\frac{1}{f_{n,a} * P_0} + \frac{1}{S_{cyt}^{A\rightarrow B} * P_{cyt}}\right) + \frac{1}{P_0 * 24 * f_{n,a}}}$$

$$= \frac{1}{\left(\frac{P_0 * 24 * f_{n,cyt} + P_{pgp}}{P_0 * 24 * f_{n,cyt}}\right) * \left(\frac{1}{f_{n,a} * P_0} + \frac{1}{S_{cyt}^{A\rightarrow B} * P_{cyt}}\right) + \frac{1}{P_0 * 24 * f_{n,a}}}$$

$$= \frac{1}{\left(\frac{1}{\frac{P_0 * 24 * f_{n,cyt}}{P_0 * 24 * f_{n,cyt} + P_{pgp}}}\right) * \left(\frac{1}{f_{n,a} * P_0} + \frac{1}{S_{cyt}^{A\rightarrow B} * P_{cyt}}\right) + \frac{1}{P_0 * 24 * f_{n,a}}} \quad (S10)$$

The permeability through the cell layer in the B ⟶ A direction can be described as follows:

$$P_{trans,B\to A} = \frac{\left(1 + \frac{P_{pgp}}{f_{n,cyt} * P_0 * 24}\right) * \frac{f_{n,b}}{f_{n,a}}}{\left(1 + \frac{P_{pgp}}{P_0 * 24 * f_{n,cyt}}\right) * \left(\frac{1}{f_{n,a} * P_0} + \frac{1}{\frac{f_{n,a}}{f_{n,cyt}} * P_{cyt}}\right) + \frac{1}{P_0 * 24 * f_{n,a}}}$$

$$= \left(1 + \frac{P_{pgp}}{f_{n,cyt} * P_0 * 24}\right) * \frac{f_{n,b}}{f_{n,a}} * P_{trans,A\to B} \qquad (S11)$$

Therefore, the full flux equation for transport in the direction A ⟶ B is defined as follows:

$$J_{A\to B} = \frac{1}{\frac{1}{\frac{(P_{trans,A\to B} + P_{para})}{(P_{trans,B\to A} + P_{para})} * P_{ABL,b}} + \frac{1}{\frac{(P_{trans,A\to B} + P_{para})}{(P_{trans,B\to A} + P_{para})} * P_{filter}} + \frac{1}{(P_{trans,A\to B} + P_{para})} + \frac{1}{P_{ABL,a}}} * C_a$$

$$- \frac{\frac{(P_{trans,B\to A} + P_{para})}{(P_{trans,A\to B} + P_{para})}}{\frac{1}{\frac{(P_{trans,A\to B} + P_{para})}{(P_{trans,B\to A} + P_{para})} * P_{ABL,b}} + \frac{1}{\frac{(P_{trans,A\to B} + P_{para})}{(P_{trans,B\to A} + P_{para})} * P_{filter}} + \frac{1}{(P_{trans,a\to b} + P_{para})} + \frac{1}{P_{ABL,a}}} * C_b \qquad (S12)$$

When expressed in the experimentally obtained metrics, Equation S12 is equivalent to:

$$J_{A\to B} = P_{app,A\to B} * C_a - P_{app,B\to A} * C_b \qquad (S13)$$

And at infinite sink conditions (C_b=0), Eq. S12 simplifies to the following:

$$J_{A\to B} = \frac{1}{\frac{1}{\frac{(P_{trans,A\to B} + P_{para})}{(P_{trans,B\to A} + P_{para})} * P_{ABL,b}} + \frac{1}{\frac{(P_{trans,A\to B} + P_{para})}{(P_{trans,B\to A} + P_{para})} * P_{filter}} + \frac{1}{(P_{trans,A\to B} + P_{para})} + \frac{1}{P_{ABL,a}}} * C_a$$

$$= \frac{1}{\frac{1}{S_{ABL,b}^{A\to B} * P_{ABL,b}} + \frac{1}{S_{ABL,b}^{A\to B} * P_{filter}} + \frac{1}{(P_{trans,A\to B} + P_{para})} + \frac{1}{P_{ABL,a}}} * C_a \qquad (S14)$$

Likewise, the full flux equation for transport in the direction B ⟶ A is defined as follows:

$$J_{B\to A} = -\frac{1}{\frac{1}{\frac{(P_{trans,A\to B} + P_{para})}{(P_{trans,B\to A} + P_{para})} * P_{ABL,b}} + \frac{1}{\frac{(P_{trans,A\to B} + P_{para})}{(P_{trans,B\to A} + P_{para})} * P_{filter}} + \frac{1}{(P_{trans,A\to B} + P_{para})} + \frac{1}{P_{ABL,a}}} * C_a$$

$$+ \frac{\frac{(P_{trans,B\to A} + P_{para})}{(P_{trans,A\to B} + P_{para})}}{\frac{1}{\frac{(P_{trans,A\to B} + P_{para})}{(P_{trans,B\to A} + P_{para})} * P_{ABL,b}} + \frac{1}{\frac{(P_{trans,A\to B} + P_{para})}{(P_{trans,B\to A} + P_{para})} * P_{filter}} + \frac{1}{(P_{trans,A\to B} + P_{para})} + \frac{1}{P_{ABL,a}}} * C_b \qquad (S15)$$

And at infinite sink conditions (C_a=0), Eq. S15 simplifies to the following:

$$J_{B\to A} = \frac{\frac{P_{trans,B\to A} + P_{para}}{P_{trans,A\to B} + P_{para}}}{\frac{1}{\frac{(P_{trans,A\to B} + P_{para})}{(P_{trans,B\to A} + P_{para})} * P_{ABL,b}} + \frac{1}{\frac{(P_{trans,A\to B} + P_{para})}{(P_{trans,B\to A} + P_{para})} * P_{filter}} + \frac{1}{(P_{trans,A\to B} + P_{para})} + \frac{1}{P_{ABL,a}}} * C_b$$

$$= \frac{1}{\frac{1}{P_{ABL,b}} + \frac{1}{P_{filter}} + \frac{1}{(P_{trans,B\to A} + P_{para})} + \frac{1}{P_{ABL,a} * \frac{(P_{trans,B\to A} + P_{para})}{(P_{trans,A\to B} + P_{para})}}} * C_b$$

$$= \frac{1}{\frac{1}{P_{ABL,b}} + \frac{1}{P_{filter}} + \frac{1}{(P_{trans,B\to A} + P_{para})} + \frac{1}{P_{ABL,a} * S_{ABL,a}^{B\to A}}} * C_b \qquad \text{(S17)}$$

As per Eq. 9 in the main text, the following relationships are derived for the ER:

$$ER = \frac{(P_{trans,B\to A} + P_{para})}{(P_{trans,A\to B} + P_{para})}$$

$$= \frac{\frac{\left(1 + \frac{P_{pgp}}{f_{n,cyt} * P_0 * 24}\right) * \frac{f_{n,b}}{f_{n,a}}}{\left(1 + \frac{P_{pgp}}{P_0 * 24 * f_{n,cyt}}\right) * \left(\frac{1}{f_{n,a} * P_0} + \frac{1}{\frac{f_{n,a}}{f_{n,cyt}} * P_{cyt}}\right) + \frac{1}{P_0 * 24 * f_{n,a}}} + P_{para}}{\frac{1}{\left(1 + \frac{P_{pgp}}{P_0 * 24 * f_{n,cyt}}\right) * \left(\frac{1}{f_{n,a} * P_0} + \frac{1}{\frac{f_{n,a}}{f_{n,cyt}} * P_{cyt}}\right) + \frac{1}{P_0 * 24 * f_{n,a}}} + P_{para}}$$

$$= \frac{\dfrac{\left(1+\dfrac{P_{pgp}}{f_{n,cyt}*P_0*24}\right)*\dfrac{f_{n,b}}{f_{n,a}}}{\left(1+\dfrac{P_{pgp}}{P_0*24*f_{n,cyt}}\right)*\left(\dfrac{1}{f_{n,a}*P_0}+\dfrac{1}{S_{cyt}^{A\to B}*P_{cyt}}\right)+\dfrac{1}{P_0*24*f_{n,a}}}+P_{para}}{\dfrac{1}{\left(1+\dfrac{P_{pgp}}{P_0*24*f_{n,cyt}}\right)*\left(\dfrac{1}{f_{n,a}*P_0}+\dfrac{1}{S_{cyt}^{A\to B}*P_{cyt}}\right)+\dfrac{1}{P_0*24*f_{n,a}}}+P_{para}} \tag{S18}$$

In the special case where P_{para} is negligible, then Equation S16 reduces to:

$$ER = \left(1+\frac{P_{pgp}}{f_{n,cyt}*P_0*24}\right)*\frac{f_{n,b}}{f_{n,a}} \tag{S19}$$

And in the special case where P_{para} is negligible and the iso-method is applied (assumed for aim i), then Eq. S19 reduces to Eq. 10 in the main text. In the special case where P_{para} dominates flux in both the A⟶B and B⟶A direction, then Eq. S18 reduces to unity.

As per Dahley *et al*. [23], the concentration shift factors used in the above equations are defined as follows:

$$S_{cyt}^{A\to B} = \frac{f_{n,a}}{f_{n,cyt}} \tag{S20}$$

$$S_{ABL,b}^{A\to B} = \frac{(P_{trans,A\to B}+P_{para})}{(P_{trans,B\to A}+P_{para})} = \frac{1}{S_{ABL,a}^{B\to A}} \tag{S21}$$

$$S_{ABL,a}^{B\to A} = \frac{(P_{trans,B\to A}+P_{para})}{(P_{trans,A\to B}+P_{para})} \tag{S22}$$

Calculation of neutral fraction (f_n)

Cation:

$$f_n = \frac{1}{10^{pK_{a1}-pH} + 1} \qquad \text{(S23)}$$

Dication:

$$f_n = \frac{1}{10^{pK_{a1}+pK_{a2}-2\cdot pH} + 10^{pK_{a1}-pH} + 1} \qquad \text{(S24)}$$

Anion:

$$f_n = \frac{1}{10^{-pK_{a1}+pH} + 1} \qquad \text{(S25)}$$

For more complex speciation, see [35].

II Chemicals

Table S2: List of chemicals and suppliers.

Chemical	Supplier
**Cell Culture**	
Dulbecco's modified Eagle medium (DMEM); high glucose, GlutaMAX™ supplement	Life Technologies Ltd., Paisley, UK
Penicillin–streptomycin<br>[+] 10,000 units/mL penicillin<br>[+] 10,000 µg/mL streptomycin	Life Technologies Corporation, Grand Island, NY, USA
Fetal bovine serum (FBS)	Life Technologies Corporation, Grand Island, NY, USA
**Monolayer Efflux Studies**	
HPS/HPU/LPS assays	
Hanks' balanced salts solution (HBSS);<br>w/ calcium, magnesium, and sodium bicarbonate	Biowest SAS, Nuaillé, France
4-Morpholineethanesulfonic acid (MES)	Sigma-Aldrich, Co., St. Louis, MO, USA
N-(2-Hydroxyethyl)piperazine-N’-(2-ethanesulfonic acid) (HEPES)	Sigma-Aldrich, Co., St. Louis, MO, USA
Lucifer Yellow CH dilithium salt	Sigma-Aldrich, Co., St. Louis, MO, USA
Dipyridamole	Alfa Aesar, Haverhill, MA, USA
Quinidine	Sigma-Aldrich, Co., St. Louis, MO, USA
Loperamide hydrochloride	Sigma-Aldrich, Co., St. Louis, MO, USA
Reference compound	
Acebutolol hydrochloride	Sigma-Aldrich, Co., St. Louis, MO, USA
P_{para} *and* P_o *assays*	
Elacridar (GF120918)	Sigma-Aldrich, Co., St. Louis, MO, USA
DMSO	Th. Geyer GmbH & Co. KG, Renningen, Germany
**Measurement of ABL thickness**	
Testosterone	Fluka, Honeywell International, Inc., NJ, USA

III Measurement of the Reference Compound

In order to ensure P-gp activity and consistency of expression and/or activity between passages and experiments, additional assays were performed with a reference compound in tandem with the assays for each compound outlined in the main text. These assays were performed in both directions, as described in the Methods section of the main text. Acebutol (15 µM) was selected as the reference compound due to its favourable price, stability, high ER, good recovery, and reliable analysis with LC/MS. Details and the results of these assays can be found below in Table S3. P_{app} represents the recovery-corrected mean values for at least three timesteps per replicate ± standard deviation, with one replicate performed in each direction.

Table S3: P_{app} and ER values for the reference compound.

**Compound**	**Associated assay compound**	**Cell passage**	$P_{app, A \rightarrow B}$ [x 10^{-6} cm/s]	$P_{app, B \rightarrow A}$ [x 10^{-6} cm/s]	**ER**	**Recovery** (%)
**Acebutolol** (15 µM)	Quinidine	29	0.26 ± 0.0	7.6 ± 1.5	28.4	99-117
	Loperamide	25	0.33 ± 0.0	8.5 ± 0.1	26.2	97-100
	Dipyridamole	30	0.37 ± 0.0	8.6 ± 0.3	23.2	98-102

IV Measurement of apparent ABL thickness

Transport assays in the A⟶B direction were performed as described in the methods section of the main text. Testosterone (1 µg/mL, 0.01% DMSO) was used as a non-substrate chemical with relatively high hydrophobicity ($logK_{hex/water}$: 0.55) in order to ensure ABL and/or filter limitation. HPS, HPU, and LPS conditions were tested for both Corning and CellQart filters. Samples were taken every 15 min for 45 min. Table S4 shows the P_{app} values and resultant calculated ABL thickness for each condition and filter brand. P_{app} represents the recovery-corrected mean values for at least three timesteps for one replicate ± standard deviation.

Table S4: Experimental details, P_{app}, and calculated ABL thickness

Filter	Porosity [pores/cm²]	Stirring [rpm]	$P_{app, A\longrightarrow B}$ [x 10^{-6} cm/s]	Recovery [%]	ABL thickness[a] [cm]
**Corning** (New York, USA: pore size: 0.4 µm; filter thickness: 11.5 µm)	100 x 10^6	450	160 ± 15.9	97	0.042
	100 x 10^6	None	57.4 ± 4.13	82	0.127
	4 x 10^6	450	53.2 ± 4.85	85	0.138
**CellQART** (Northeim, Germany: pore size: 0.4 µm; filter thickness: 11.5 µm)	100 x 10^6	450	158 ± 5.99	92	0.043
	100 x 10^6	None	51.4 ± 6.33	79	0.143
	2 x 10^6	450	21.1 ± 1.58	79	0.358

a refers to the total ABL thickness (apical and basolateral) and includes filter resistance. In the case of filter limitation, this refers to "apparent" ABL thickness due to the resistance of the water-filled pores.

Based on these results, it is clear that the brand of filter used and the porosity as well as the shaking conditions significantly affect the apparent ABL thickness. For the ideal system (HPS, pH 7.4), neutral compounds with a $logK_{hex/water}$ in the order of > -4 are expected to be ABL-limited in at least one direction, whereas for low porosity filter systems and non-shaking conditions, the $logP_{app}$ shifts by 0.5–1 log units, and chemicals with a $logK_{hex/water}$ in the order of > -5 can be ABL-limited in at least one direction.

V Measurement of P_0

It is well known that membrane permeability, P_m, can only be extracted from P_{app} if P_{app} is not dominated by diffusion through the ABL and filter, and not dominated by paracellular transport. Dahley et al. [23] recently detailed methods to ensure that ABL and filter effects are excluded when determining membrane permeability P_m.

Where possible, additional assays were performed in tandem with the experiments outlined in the main text to measure the P_0. These assays were performed as described in the Methods section of the main text; however, stock solutions with the compounds under investigation were prepared with the addition of the P-gp inhibitor elacridar (2 µM) to ensure that no active efflux of these substrates would affect the measured transport rates. Due to its poor aqueous solubility, elacridar was prepared in DMSO. However, it was ensured that the final DMSO concentration of the stock solution was 0.01 %, thereby avoiding any potential effects of DMSO on the transporter [37]. Consequently, 0.01 % of DMSO was added to the transport buffer to ensure that no gradient effects would influence the assay results. Additionally, assays may have been performed at various other pH values (iso-pH method) to ensure the avoidance of ABL/filter limitation. Details and the results of these assays can be found below in Table S5. P_{app} represents the recovery-corrected mean values for at least three timesteps per replicate ± standard deviation, with one replicate performed in each direction.

Table S5: P_{app} with the inhibitor, ER, and calculated $\log P_o$ for the measured compounds

Compound	pH	$P_{app, A\rightarrow B}$ [x 10^{-6} cm/s]	$P_{app, B\rightarrow A}$ [x 10^{-6} cm/s]	ER	Recovery [%]	pk_a [a]	f_n [%]	$\log P_0$ [b]
**Loperamide** (10 µM) + **Elacridar**	6.5	88.8 ± 3.9	90.1 ± 3.7	1.0	60 - 80	8.46	1.80	> -1.9[c]
**Quinidine** (10 µM) + **Elacridar**	6.0	32.1 ± 2.1	29.5 ± 2.5	0.9	92-103	8.31	0.48	-2.0
**Dipyridamole** (12µM) + **Elacridar**	9	22.3 ± 1.5	34.5 ± 8.9	1.5	98-113	4.89	99.9	-4.5

[a] Determined at 37°C, from Avdeef [31]

[b] Calculated from $P_{app, A\rightarrow B}$

[c] ABL-limited P_{app} and the consequent extraction of P_0 is not possible; therefore, the value represents the lower limit of compound P_0

VI Detailed derivation of P_{trans}

Eq.		Explanation
A1	$J_{trans,A\to B} = c_{ABL,a} * f_{n,a} * P_0 * 24 - c_{cyt,a} * \left(f_{n,cyt} * P_0 * 24 + P_{pgp}\right) = P_{cyt} * \left(c_{cyt,a} - c_{cyt,b}\right) = c_{cyt,b} * f_{n,cyt} * P_0 - c_{filter} * f_{n,b} * P_0$	*Equations S6–S8 in S9*
A2	$P_{cyt} * \left(c_{cyt,a} - c_{cyt,b}\right) = c_{cyt,b} * f_{n,cyt} * P_0 - c_{filter} * f_{n,b} * P_0$	*Last part of A1,* $J_{cyt} = J_{m,b}$
A3	$c_{cyt,b} = \frac{c_{filter} * f_{n,b} * P_0 + P_{cyt} * c_{cyt,a}}{P_{cyt} + f_{n,cyt} * P_0}$	*Solve A2 for* $c_{cyt,b}$
A4	$c_{ABL,a} * f_{n,a} * P_0 * 24 - c_{cyt,a} * \left(f_{n,cyt} * P_0 * 24 + P_{pgp}\right) = P_{cyt} * \left(c_{cyt,a} - c_{cyt,b}\right)$	*First part of A1,* $J_{m,a} = J_{cyt}$
A5	$c_{ABL,a} * f_{n,a} * P_0 * 24 - c_{cyt,a} * \left(f_{n,cyt} * P_0 * 24 + P_{pgp}\right) = P_{cyt} * \left(c_{cyt,a} - \frac{c_{filter} * f_{n,b} * P_0 + P_{cyt} * c_{cyt,a}}{P_{cyt} + f_{n,cyt} * P_0}\right)$	*A3 in A4*
A6	$c_{ABL,a} * f_{n,a} * P_0 * 24 - c_{cyt,a} * \left(f_{n,cyt} * P_0 * 24 + P_{pgp}\right) = P_{cyt} * \left(\frac{f_{n,cyt} * P_0 * c_{cyt,a} - c_{filter} * f_{n,b} * P_0}{P_{cyt} + f_{n,cyt} * P_0}\right)$	*Rearrange A5*
A7	$c_{cyt,a} = \frac{c_{ABL,a} * f_{n,a} * P_0 * 24 + \frac{P_{cyt} * c_{filter} * f_{n,b} * P_0}{P_{cyt} + f_{n,cyt} * P_0}}{\left(f_{n,cyt} * P_0 * 24 + P_{pgp}\right) + \frac{P_{cyt} * f_{n,cyt} * P_0}{P_{cyt} + f_{n,cyt} * P_0}}$	*Solve A6 for* $c_{cyt,a}$
A8	$c_{cyt,a} = \frac{c_{ABL,a} * f_{n,a} * P_0 * 24 * (P_{cyt} + f_{n,cyt} * P_0) + P_{cyt} * c_{filter} * f_{n,b} * P_0}{(P_{cyt} + f_{n,cyt} * P_0) * \left(f_{n,cyt} * P_0 * 24 + P_{pgp}\right) + P_{cyt} * f_{n,cyt} * P_0}$	*Rearrange A7*
A9	$J_{trans,A\to B} = c_{ABL,a} * f_{n,a} * P_0 * 24 - c_{cyt,a} * \left(f_{n,cyt} * P_0 * 24 + P_{pgp}\right)$	*First part of A1,* $J_{trans,A\to B} = J_{m,a} = J_{cyt}$
A10	$J_{trans,A\to B} = c_{ABL,a} * f_{n,a} * P_0 * 24 - \frac{c_{ABL,a} * f_{n,a} * P_0 * 24 * (P_{cyt} + f_{n,cyt} * P_0) + P_{cyt} * c_{filter} * f_{n,b} * P_0}{(P_{cyt} + f_{n,cyt} * P_0) * \left(f_{n,cyt} * P_0 * 24 + P_{pgp}\right) + P_{cyt} * f_{n,cyt} * P_0} * \left(f_{n,cyt} * P_0 * 24 + P_{pgp}\right)$	*A8 in A9*
A11	$J_{trans,A\to B} = \frac{c_{ABL,a} * f_{n,a} * P_0 * 24 * \left((P_{cyt} + f_{n,cyt} * P_0) * \left(f_{n,cyt} * P_{P_0*24} + P_{pgp}\right) + P_{cyt} * f_{n,cyt} * P_0\right) - c_{ABL,a} * f_{n,a} * P_0 * 24 * (P_{cyt} + f_{n,cyt} * P_0) * \left(f_{n,cyt} * P_0 * 24 + P_{pgp}\right) - P_{cyt} * c_{filter} * f_{n,b} * P_0 * \left(f_{n,cyt} * P_0 * 24 + P_{pgp}\right)}{(P_{cyt} + f_{n,cyt} * P_0) * \left(f_{n,cyt} * P_0 * 24 + P_{pgp}\right) + P_{cyt} * f_{n,cyt} * P_0}$	*Rearrange A10*
A12	$J_{trans,A\to B} = \frac{c_{ABL,a} * f_{n,a} * P_0 * 24 * \left(P_{cyt} * f_{n,cyt} * P_0\right) - P_{cyt} * c_{filter} * f_{n,b} * P_0 * \left(f_{n,cyt} * P_0 * 24 + P_{pgp}\right)}{(P_{cyt} + f_{n,cyt} * P_0) * \left(f_{n,cyt} * P_0 * 24 + P_{pgp}\right) + P_{cyt} * f_{n,cyt} * P_0}$	*Rearrange*
A13	$J_{trans,A\to B} = \frac{f_{n,a} * P_0 * 24 * \left(P_{cyt} * f_{n,cyt} * P_0\right)}{(P_{cyt} + f_{n,cyt} * P_0) * \left(f_{n,cyt} * P_0 * 24 + P_{pgp}\right) + P_{cyt} * f_{n,cyt} * P_0} * c_{ABL,a} - \frac{P_{cyt} * f_{n,b} * P_0 * \left(f_{n,cyt} * P_0 * 24 + P_{pgp}\right)}{(P_{cyt} + f_{n,cyt} * P_0) * \left(f_{n,cyt} * P_0 * 24 + P_{pgp}\right) + P_{cyt} * f_{n,cyt} * P_0} * c_{filter}$	*Rearrange*

A14	$J_{trans,A\to B} = \frac{f_{n,a} * P_0 * 24 * P_{cyt} * f_{n,cyt} * P_0}{P_{cyt} * f_{n,cyt} * P_0 * 24 + f_{n,cyt} * P_0 * f_{n,cyt} * P_0 * 24 + P_{cyt} * P_{pgp} + f_{n,cyt} * P_0 * P_{pgp} + P_{cyt} * f_{n,cyt} * P_0} * c_{ABL,a} - \frac{P_{cyt} * f_{n,b} * P_0 * f_{n,cyt} * P_{ma} + P_{cyt} * f_{n,b} * P_0 * P_{pgp}}{P_{cyt} * f_{n,cyt} * P_0 * 24 + f_{n,cyt} * P_0 * f_{n,cyt} * P_0 * 24 + P_{cyt} * P_{pgp} + f_{n,cyt} * P_0 * P_{pgp} + P_{cyt} * f_{n,cyt} * P_0} * c_{filter}$	*Rearrange*
A15	$J_{trans,A\to B} = \frac{f_{n,a} * P_0 * 24 * P_{cyt} * f_{n,cyt} * P_0}{P_{cyt} * f_{n,cyt} * P_0 * 24 + f_{n,cyt} * P_0 * f_{n,cyt} * P_0 * 24 + P_{cyt} * P_{pgp} + f_{n,cyt} * P_0 * P_{pgp} + P_{cyt} * f_{n,cyt} * P_0} * c_{ABL,a} - \frac{f_{n,a} * P_0 * 24 * P_{cyt} * f_{n,cyt} * P_0 * \frac{f_{n,b}}{f_{n,a}}(1 + \frac{P_{pgp}}{f_{n,cyt} * P_0 * 24})}{P_{cyt} * f_{n,cyt} * P_0 * 24 + f_{n,cyt} * P_0 * f_{n,cyt} * P_0 * 24 + P_{cyt} * P_{pgp} + f_{n,cyt} * P_0 * P_{pgp} + P_{cyt} * f_{n,cyt} * P_0} * c_{filter}$	*Rearrange*
A16	$J_{trans,A\to B} = \frac{1}{\frac{1}{f_{n,a} * P_0} + \frac{1}{P_{cyt} * f_{n,a}/f_{n,cyt}} + \frac{P_{pgp}}{f_{n,a} * P_0 * 24 * f_{n,cyt} * P_0} + \frac{P_{pgp}}{P_{cyt} * f_{n,a} * P_0 * 24 *} + \frac{1}{f_{n,a} * P_0 * 24}} * c_{ABL,a} - \frac{\frac{f_{n,b}}{f_{n,a}}(1 + \frac{P_{pgp}}{f_{n,cyt} * P_0 * 24})}{\frac{1}{f_{n,a} * P_0} + \frac{1}{P_{cyt} * f_{n,a}/f_{n,cyt}} + \frac{P_{pgp}}{f_{n,a} * P_0 * 24 * f_{n,cyt} * P_0} + \frac{P_{pgp}}{P_{cyt} * f_{n,a} * P_0 * 24 *} + \frac{1}{f_{n,a} * P_0 * 24}} * c_{filter}$	*Rearrange*
A17	$J_{trans,A\to B} = \frac{1}{\frac{1}{f_{n,a} * P_0 * 24} + \left(1 + \frac{P_{pgp}}{P_0 * 24 * f_{n,cyt}}\right) * (\frac{1}{\frac{f_{n,a}}{f_{n,cyt}} * P_{cyt}} + \frac{1}{f_{n,a} * P_0}} * c_{ABL,a} - \frac{1}{\frac{1}{f_{n,a} * P_0 * 24} + \left(1 + \frac{P_{pgp}}{P_0 * 24 * f_{n,cyt}}\right) * \left(\frac{1}{\frac{f_{n,a}}{f_{n,cyt}} * P_{cyt}} + \frac{1}{f_{n,a} * P_0}\right)} * \frac{f_{n,b}}{f_{n,a}}(1 + \frac{P_{pgp}}{f_{n,cyt} * P_0 * 24}) * c_{filter}$	*Rearrange*
A18	$J_{trans,A\to B} = P_{trans,A\to B} * c_{ABL,a} - P_{trans,B\to A} * c_{filter}$	
A19	$P_{trans,A\to B} = \frac{1}{\frac{1}{f_{n,a} * P_0 * 24} + \left(1 + \frac{P_{pgp}}{P_0 * 24 * f_{n,cyt}}\right) * (\frac{1}{\frac{f_{n,a}}{f_{n,cyt}} * P_{cyt}} + \frac{1}{f_{n,a} * P_0})}$	$P_{trans,A\to B}\ from\ A17$

A20	$$P_{trans,B\to A} = \frac{1}{\frac{1}{f_{n,a} * P_0 * 24} + \left(1 + \frac{P_{pgp}}{P_0 * 24 * f_{n,cyt}}\right) * \left(\frac{1}{\frac{f_{n,a}}{f_{n,cyt}} * P_{cyt}} + \frac{1}{f_{n,a} * P_0}\right)} * \frac{f_{n,b}}{f_{n,a}}\left(1 + \frac{P_{pgp}}{f_{n,cyt} * P_0 * 24}\right) = \frac{f_{n,b}}{f_{n,a}}\left(1 + \frac{P_{pgp}}{f_{n,cyt} * P_0 * 24}\right) * P_{trans,A\to B}$$	$P_{trans,B\to A}\ from\ A17$

VII Detailed derivation of total flux

Eq.		Explanation
B1	$$J_{A\to B} = J_{ABL,a} = J_{cell} = J_{filter} = J_{ABL,b}$$	*Equation S6*
B2	$$P_{filter}(C_{filter} - C_{ABL,b}) = P_{ABL,b}(C_{ABL,b} - C_b)$$	$J_{filter} = J_{ABL,b}$
B3	$$C_{ABL,b} = \frac{P_{filter} * C_{filter} + P_{ABL,b} * C_b}{P_{filter} + P_{ABL,b}}$$	*Solve B2 for* $C_{ABL,b}$
B4	$$P_{trans,A\to B} * C_{ABL,a} - P_{trans,B\to A} * C_{filter} + P_{para} * (C_{ABL,a} - C_{filter}) = P_{filter}(C_{filter} - C_{ABL,b})$$	$J_{cell} = J_{filter}$
B5	$$P_{trans,A\to B} * C_{ABL,a} - P_{trans,B\to A} * C_{filter} + P_{para} * (C_{ABL,a} - C_{filter}) = P_{filter}\left(C_{filter} - \frac{P_{filter} * C_{filter} + P_{ABL,b} * C_b}{P_{filter} + P_{ABL,b}}\right)$$	*Insert B3 into B4*
B6	$$(P_{trans,A\to B} + P_{para}) * C_{ABL,a} - (P_{trans,B\to A} + P_{para}) * C_{filter} = P_{filter}\left(\frac{P_{ABL,b} * C_{filter} - P_{ABL,b} * C_b}{P_{filter} + P_{ABL,b}}\right)$$	*Rearrange B5*
B7	$$C_{filter} = \frac{(P_{trans,A\to B} + P_{para}) * C_{ABL,a} + \frac{P_{filter} * P_{ABL,b} * C_b}{P_{filter} + P_{ABL,b}}}{(P_{trans,B\to A} + P_{para}) + \frac{P_{filter} * P_{ABL,b}}{P_{filter} + P_{ABL,b}}}$$	*Solve B6 for* C_{filter}
B8	$$P_{ABL,a}(C_a - C_{ABL,a}) = (P_{trans,A\to B} + P_{para}) * C_{ABL,a} - (P_{trans,B\to A} + P_{para}) * C_{filter}$$	$J_{A\to B} = J_{ABL,a}$

B9	$P_{ABL,a}(C_a - C_{ABL,a}) = (P_{trans,A\to B} + P_{para}) * C_{ABL,a} - (P_{trans,B\to A} + P_{para}) * \frac{(P_{trans,A\to B} + P_{para}) * C_{ABL,a} + \frac{P_{filter} * P_{ABL,b} * C_b}{P_{filter} + P_{ABL,b}}}{(P_{trans,B\to A} + P_{para}) + \frac{P_{filter} * P_{ABL,b}}{P_{filter} + P_{ABL,b}}}$	*Insert B7 into B8*
B10	$P_{ABL,a}(C_a - C_{ABL,a}) = \frac{(P_{trans,A\to B} + P_{para}) * \frac{P_{filter} * P_{ABL,b}}{P_{filter} + P_{ABL,b}} * C_{ABL,a} - (P_{trans,B\to A} + P_{para}) * \frac{P_{filter} * P_{ABL,b}}{P_{filter} + P_{ABL,b}} * C_b}{(P_{trans,B\to A} + P_{para}) + \frac{P_{filter} * P_{ABL,b}}{P_{filter} + P_{ABL,b}}}$	*Rearrange B9*
B11	$C_{ABL,a} = \frac{P_{ABL,a} * C_a + \frac{(P_{trans,B\to A} + P_{para}) * \frac{P_{filter} * P_{ABL,b}}{P_{filter} + P_{ABL,b}} * C_b}{(P_{trans,B\to A} + P_{para}) + \frac{P_{filter} * P_{ABL,b}}{P_{filter} + P_{ABL,b}}}}{P_{ABL,a} + \frac{(P_{trans,A\to B} + P_{para}) * \frac{P_{filter} * P_{ABL,b}}{P_{filter} + P_{ABL,b}}}{(P_{trans,B\to A} + P_{para}) + \frac{P_{filter} * P_{ABL,b}}{P_{filter} + P_{ABL,b}}}}$	*Solve B10 for* $C_{ABL,a}$
B12	$J_{A\to B} = P_{ABL,a}(C_a - C_{ABL,a})$	
B13	$J_{A\to B} = P_{ABL,a}\left(C_a - \frac{P_{ABL,a} * C_a + \frac{(P_{trans,B\to A} + P_{para}) * \frac{P_{filter} * P_{ABL,b}}{P_{filter} + P_{ABL,b}} * C_b}{(P_{trans,B\to A} + P_{para}) + \frac{P_{filter} * P_{ABL,b}}{P_{filter} + P_{ABL,b}}}}{P_{ABL,a} + \frac{(P_{trans,A\to B} + P_{para}) * \frac{P_{filter} * P_{ABL,b}}{P_{filter} + P_{ABL,b}}}{(P_{trans,B\to A} + P_{para}) + \frac{P_{filter} * P_{ABL,b}}{P_{filter} + P_{ABL,b}}}}\right)$	*Insert B11 into B12*
B14	$J_{A\to B} = P_{ABL,a}\left(\frac{\frac{(P_{trans,A\to B} + P_{para}) * \frac{P_{filter} * P_{ABL,b}}{P_{filter} + P_{ABL,b}}}{(P_{trans,B\to A} + P_{para}) + \frac{P_{filter} * P_{ABL,b}}{P_{filter} + P_{ABL,b}}} * C_a - \frac{(P_{trans,B\to A} + P_{para}) * \frac{P_{filter} * P_{ABL,b}}{P_{filter} + P_{ABL,b}} * C_b}{(P_{trans,B\to A} + P_{para}) + \frac{P_{filter} * P_{ABL,b}}{P_{filter} + P_{ABL,b}}}}{P_{ABL,a} + \frac{(P_{trans,A\to B} + P_{para}) * \frac{P_{filter} * P_{ABL,b}}{P_{filter} + P_{ABL,b}}}{(P_{trans,B\to A} + P_{para}) + \frac{P_{filter} * P_{ABL,b}}{P_{filter} + P_{ABL,b}}}}\right)$	*Rearrange*
B15	$J_{A\to B} = P_{ABL,a}\left(\frac{(P_{trans,A\to B} + P_{para}) * \frac{P_{filter} * P_{ABL,b}}{P_{filter} + P_{ABL,b}} * C_a - (P_{trans,B\to A} + P_{para}) * \frac{P_{filter} * P_{ABL,b}}{P_{filter} + P_{ABL,b}} * C_b}{\left((P_{trans,B\to A} + P_{para}) + \frac{P_{filter} * P_{ABL,b}}{P_{filter} + P_{ABL,b}}\right) * P_{ABL,a} + (P_{trans,A\to B} + P_{para}) * \frac{P_{filter} * P_{ABL,b}}{P_{filter} + P_{ABL,b}}}\right)$	*Rearrange*

B16	$J_{A\rightarrow B} = P_{ABL,a}\left(\frac{(P_{trans,A\rightarrow B}+P_{para})*P_{filter}*P_{ABL,b}*C_a}{((P_{trans,B\rightarrow A}+P_{para})*(P_{filter}+P_{ABL,b}))+P_{filter}*P_{ABL,b})*P_{ABL,a}+(P_{trans,A\rightarrow B}+P_{para})*P_{filter}*P_{ABL,b}}\right)$- $P_{ABL,a}\left(\frac{\left(P_{trans,B\rightarrow A}+P_{para}\right)*P_{filter}*P_{ABL,b}*C_b}{\left(\left(P_{trans,B\rightarrow A}+P_{para}\right)*(P_{filter}+P_{ABL,b})+P_{filter}*P_{ABL,b}\right)*P_{ABL,a}+(P_{trans,A\rightarrow B}+P_{para})*P_{filter}*P_{ABL,b}}\right)$	*Rearrange*
B17	$J_{A\rightarrow B} = \frac{1}{\frac{1}{\frac{P_{trans,A\rightarrow B}+P_{para}}{P_{trans,B\rightarrow A}+P_{para}}}*(\frac{1}{P_{filter}}+\frac{1}{P_{ABL,b}})+\frac{1}{P_{trans,A\rightarrow B}+P_{para}}+\frac{1}{P_{ABL,a}}}*C_a$- $\frac{1}{\frac{1}{P_{filter}}+\frac{1}{P_{ABL,b}}+\frac{1}{P_{trans,B\rightarrow A}+P_{para}}+\frac{1}{\frac{P_{trans,B\rightarrow A}+P_{para}}{P_{trans,A\rightarrow B}+P_{para}}}*\frac{1}{P_{ABL,a}}}*C_b$	*Rearrange*
B18	$J_{A\rightarrow B} = -J_{B\rightarrow A}$	
B19	$J_{B\rightarrow A} = -\frac{1}{\frac{1}{\frac{P_{trans,A\rightarrow B}+P_{para}}{P_{trans,B\rightarrow A}+P_{para}}}*(\frac{1}{P_{filter}}+\frac{1}{P_{ABL,b}})+\frac{1}{P_{trans,A\rightarrow B}+P_{para}}+\frac{1}{P_{ABL,a}}}*C_a+$ $\frac{1}{\frac{1}{P_{filter}}+\frac{1}{P_{ABL,b}}+\frac{1}{P_{trans,B\rightarrow A}+P_{para}}+\frac{1}{\frac{P_{trans,B\rightarrow A}+P_{para}}{P_{trans,A\rightarrow B}+P_{para}}}*\frac{1}{P_{ABL,a}}}*C_b$	
